# Targeted Nipple-Areola Complex (NAC) Reinnervation (TNR): A Systematic Review and Meta-Analysis

**DOI:** 10.1007/s00266-025-05179-y

**Published:** 2025-08-28

**Authors:** Antoinette T. Nguyen, Rebeka A. Dejenie, Rena A. Li, Marco F. Ellis, Robert D. Galiano

**Affiliations:** 1 School of Medicine and Dentistry, University of Rochester, Rochester, New York United States; 2 UC Davis School of Medicine, Sacramento, California United States; 3 Department of Plastic Surgery, Northwestern University Feinberg School of Medicine Chicago, 259 East Erie Street, Suite 2060, Illinois, 60611 United States

**Keywords:** Targeted nipple reinnervation, Gender-affirming surgery, Mastectomy, Nipple-areola complex, Systematic review, Meta-analysis

## Abstract

**Background:**

Loss of nipple-areola complex (NAC) sensation following mastectomy is a significant concern in both gender-affirming and oncologic breast surgery. Targeted nipple-areola complex reinnervation (TNR) has emerged as a technique to improve sensory outcomes by restoring nerve connections to the NAC. This study systematically reviews the literature and conducts a meta-analysis to evaluate the efficacy of TNR in both gender-affirming mastectomy and oncologic breast reconstruction.

**Methods:**

A systematic review and meta-analysis were conducted following PRISMA guidelines. PubMed, Embase, and Scopus were searched for studies reporting NAC neurotization outcomes. Studies were included if they assessed sensory recovery following TNR in gender-affirming mastectomy or oncologic breast reconstruction. A random-effects meta-analysis was performed on four studies reporting monofilament thresholds, with separate meta-analyses conducted for areola sensation and peripheral breast sensation. Heterogeneity between studies was assessed.

**Results:**

Twelve studies encompassing 342 participants (195 with TNR, 147 controls) were included. Meta-analysis of TNR in gender-affirming mastectomy patients demonstrated significantly improved NAC, areola, and peripheral breast skin sensation compared to controls (MD: − 1.73, 95% CI: − 2.15 to − 1.32, *p* < 0.0001, I^2^ = 67.78%; MD: − 1.73, 95% CI: − 1.91 to − 1.56, *p* < 0.0001, I^2^ = 0%; MD: − 1.59, 95% CI: − 1.81 to − 1.37, *p* < 0.0001, I^2^ = 0% respectively). Comparisons between gender-affirming and oncologic mastectomy cohorts indicated earlier and more consistent sensory recovery in gender-affirming procedures, likely due to proactive neurotization techniques.

**Conclusion:**

TNR significantly enhances NAC sensation in both gender-affirming and oncologic mastectomy patients. Gender-affirming cases demonstrate earlier and more predictable recovery, whereas oncologic reconstruction studies show more variable outcomes. Further research is needed to standardize neurotization techniques and evaluate long-term sensory restoration.

**Level of Evidence III:**

This journal requires that authors assign a level of evidence to each article. For a full description of these Evidence-Based Medicine ratings, please refer to the Table of Contents or the online Instructions to Authors www.springer.com/00266.

## Important Points

1. Targeted nipple-areola complex reinnervation (TNR) significantly improves sensory outcomes—including nipple, areola, and peripheral breast skin sensation—especially in gender-affirming mastectomy patients, as supported by meta-analysis (*p* < 0.0001 across all domains).
2. Sensory recovery is earlier and more consistent in gender-affirming mastectomy compared to oncologic reconstruction, likely due to a younger patient population and proactive nerve coaptation.
3. Oncologic breast reconstruction shows more variable and delayed outcomes, with some patients demonstrating limited or partial recovery, highlighting a need for optimized TNR protocols in this population.
4. TNR is surgically and economically feasible, adding approximately 54 minutes to operative time without significantly impacting work relative value unit (wRVU) efficiency, supporting broader implementation in both gender-affirming and oncologic contexts.

## Introduction

Loss of nipple-areola complex (NAC) sensation following mastectomy is a significant concern in both gender-affirming and oncologic breast surgery, impacting physical perception, psychological well-being, and overall patient satisfaction. The NAC plays a critical sensory and erogenous role, and its functional preservation has been an increasing focus in reconstructive surgery. [1–3] However, traditional mastectomy techniques—whether performed for gender affirmation or oncologic indications—often result in significant sensory deficits due to the disruption of intercostal nerve branches during tissue removal. [4] To mitigate these deficits, targeted nipple-areola complex reinnervation (TNR) has emerged as a technique aimed at restoring neural continuity and improving sensory recovery in post-mastectomy patients. [1–5]

Gender-affirming mastectomy, commonly referred to as “top surgery,” is one of the most frequently performed gender-affirming procedures, alleviating gender dysphoria and improving psychological and social well-being. [6] Numerous studies have demonstrated its benefits in reducing anxiety, depression, and body dissatisfaction, while enhancing psychosocial and sexual functioning. [7–11] However, a major drawback of the double-incision mastectomy with free nipple grafting (FNG)—the most common technique used for gender-affirming mastectomy—is the near-total loss of NAC sensation. Patients often report long-term sensory deficits, with many experiencing persistent numbness or reduced tactile perception postoperatively. [12–15] Given the profound impact of NAC sensation on quality of life, targeted neurotization of the NAC in gender-affirming mastectomy has gained increasing attention. TNR involves the identification, preservation, and reconnection of intercostal nerve branches to the NAC during mastectomy, thereby promoting nerve regeneration and sensory recovery. [16] While this technique has been extensively studied in the oncologic mastectomy and breast reconstruction literature, research on its application in gender-affirming surgery remains limited. [1] Understanding the efficacy of TNR in this patient population is essential, particularly given the younger age and different surgical priorities of transgender and nonbinary patients undergoing mastectomy.

In contrast to gender-affirming mastectomy, oncologic mastectomy is primarily performed for cancer treatment, with nipple-sparing mastectomy (NSM) increasingly utilized in patients with favorable tumor characteristics. [17–20] While NSM preserves the NAC anatomically, spontaneous sensory recovery remains highly variable and often incomplete. [21] Studies report that while some patients regain partial NAC sensation postoperatively, a significant proportion continue to experience numbness or dysesthesia, particularly when implant-based reconstruction is performed. [21–24] To address these sensory deficits, TNR has been integrated into oncologic breast reconstruction, particularly in the setting of autologous breast reconstruction (ABR). By reconnecting transected intercostal nerves to nerve grafts or flap-based donor nerves, targeted neurotization offers the potential for more predictable and robust sensory restoration. [25, 26] However, research on TNR in oncologic settings has produced heterogeneous results, with variations in nerve selection, surgical techniques, and sensory outcome assessment complicating direct comparisons. [4, 22, 27, 28]

While both gender-affirming and oncologic mastectomy populations share concerns regarding NAC sensory loss, key differences exist between these groups. Gender-affirming mastectomy patients often undergo surgery at younger ages, without preoperative nerve damage from radiation or prior surgery, potentially facilitating better nerve regeneration and sensory recovery. Furthermore, the surgical goals and techniques differ significantly: oncologic procedures prioritize oncologic safety and anatomical preservation, whereas gender-affirming mastectomies often involve more aggressive tissue removal and complete NAC grafting, leading to greater sensory deficits [29]

This systematic review and meta-analysis aim to synthesize the available literature on TNR across both gender-affirming and oncologic mastectomy populations, evaluate differences in sensory recovery, and identify best practices for optimizing outcomes. By examining the effectiveness of TNR in both settings, this study seeks to determine whether proactive nerve preservation and neurotization techniques can improve post-mastectomy sensory outcomes across diverse patient populations.

## Methods

A systematic review and meta-analysis were conducted to identify studies reporting patient outcomes following TNR in gender-affirming mastectomy and oncologic mastectomy. The review followed the Preferred Reporting Items for Systematic Reviews and Meta-Analyses (PRISMA) guidelines and was registered in PROSPERO (CRD 420251004025).

**Fig. 1:**
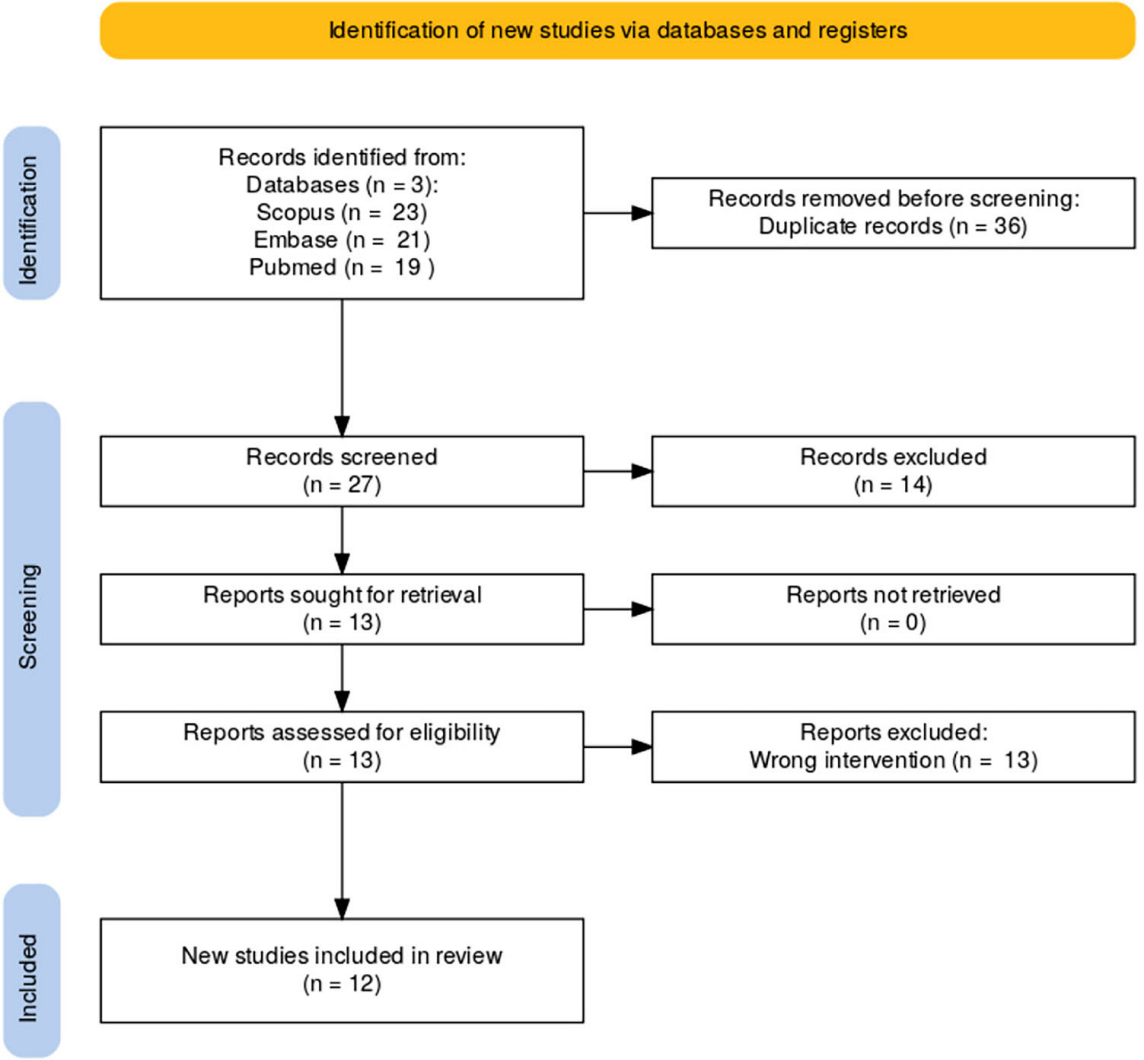
PRISMA diagram

An extensive search was performed across multiple databases including PubMed, Embase, and Scopus using the following search query: (("nipple neurotization"[Title/Abstract] OR "nipple reinnervation"[Title/Abstract] OR "targeted nipple reinnervation"[Title/Abstract] OR "NAC neurotization"[Title/Abstract] OR "nipple-areola complex neurotization"[Title/Abstract] OR "nipple-areola complex reinnervation"[Title/Abstract] OR "nipple sensation restoration"[Title/Abstract] OR "nipple sensory recovery"[Title/Abstract] OR "nipple-areola complex sensation"[Title/Abstract]). The search strategy is outlined in the PRISMA diagram (Figure 1).

Studies were included if they assessed sensory recovery following TNR in gender-affirming mastectomy or oncologic breast reconstruction. Excluded studies included those that did not focus on TNR specifically or that involved unrelated interventions. The initial search yielded 63 studies of which 36 were removed as duplicates. The remaining studies were then screened by title and abstract for relevance by two independent reviewers, further excluding 14 studies.

After full text review, 12 studies were included in the final analysis. Data were extracted by independent reviewers including study design, participant count, mean age, and outcome measures including sensory outcomes. Two independent reviewers extracted data, which was cross-checked for consistency. Risk of bias was assessed based on study design using ROBINS-I for non-randomized cohort and case-control studies, the Joanna Briggs Institute (JBI) Checklist for retrospective chart reviews. Each study was evaluated for selection bias, confounding, outcome measurement reliability, and reporting bias to determine the overall risk of bias (Table 1). Studies with high risk of bias were not excluded but were interpreted with caution

**Table 1:** Risk of bias table

Study	Study design	Risk of bias tool used	Risk of bias judgment
Remy et al. (2024)	Prospective matched cohort study	ROBINS-I	Moderate (selection bias possible due to matching)
Loughran et al. (2024)	Prospective cohort study	ROBINS-I	Moderate (potential for confounding)
Deptula & Nguyen (2021)	Retrospective chart review	JBI Checklist	High (small sample, potential for selection bias)
Remy et al. (2024)	Prospective cohort study	ROBINS-I	Moderate (potential for confounding, small sample)
Rochlin et al. (2020)	Prospective cohort	ROBINS-I	Moderate (lack of blinding, possible confounders)
Peled et al. (2023)	Prospective cohort	ROBINS-I	Low (well-controlled study, multiple sensory assessments)
Zhang et al. (2024)	Retrospective study	JBI Checklist	High (limited sample size)
Tevlin et al. (2021)	Retrospective chart review	JBI Checklist	High (limited sample size, high risk of bias in outcome assessment)
Djohan et al. (2020)	Prospectively maintained cohort (retrospective analysis)	ROBINS-I	Moderate (incomplete follow-up, retrospective component introduces bias)
Chang et al. (2024)	Retrospective case-control study	ROBINS-I	Moderate (matched contralateral breast controls improve internal validity)
Chirappapha et al. (2018)	Prospective study	ROBINS-I	Moderate (potential loss to follow-up, self-reported sensory outcomes)
Das et al. (2024)	Retrospective cross-sectional analysis	JBI Checklist	High (economic analysis, lacks patient-level outcome data)

Meta-analyses were conducted using a random effects model on four studies reporting monofilament thresholds for the NAC after TNR compared to the control in gender-affirming mastectomy. Additionally, separate meta-analyses were performed for gender-affirming mastectomy studies evaluating areola sensation and peripheral breast sensation. Heterogeneity between studies was assessed using I^2^ statistics, Cochran’s Q test, and τ^2^ estimates, and forest plots were generated for each. Statistical analyses for the meta-analyses were conducted in R (version 4.4.2) using the metafor packages.

## Results

A total of 12 studies, including 342 participants (195 who underwent TNR and 147 controls), were included in this analysis. These studies encompassed diverse methodologies, reflecting the range of surgical techniques and outcome measures utilized in both gender-affirming mastectomy and oncologic breast reconstruction (Table 2).

**Table 2:** Summary of included studies

Author	Year	Study design	Number of participants	Mean age ± SD	Surgical technique	Assessment tool	Key statistical findings	Key findings summary
Remy et al. [1]	2024	Prospective matched cohort study	50 (25 with TNR, 25 controls)	24.9 ± 5.5 years	Targeted Nipple-Areola Complex Reinnervation (TNR) during gender-affirming mastectomy	Monofilament testing, quantitative sensory testing, patient-reported outcomes	Significant improvement in sensory restoration at 12 months in TNR group vs. control (e.g., monofilament detection: NAC 3.7 ± 0.5 vs. 4.9 ± 0.9, *P* < .001; vibration: NAC 7.7 ± 0.4 vs. 7.3 ± 0.4, *P* < .001; erogenous sensation: *P* = .01)	TNR led to improved mechanical, temperature, and pain detection, as well as better erogenous sensation and patient satisfaction compared to the control group
Loughran et al.	2024	Prospective cohort study	115 patients (46 analyzed for sensory testing)	23.6 years (range 16–41)	Direct neurotization of free nipple grafts with cadaveric nerve grafts following gender-affirming mastectomy	Semmes Weinstein monofilament testing	< 1 year: 58.6% with normal to diminished sensation; >1 year: 70.8% with normal to diminished sensation	Direct neurotization improved nipple-areolar complex sensation over ^15^time, surpassing expected outcomes for standard full-thickness skin grafts.
Deptula & Nguyen [31]	2021	Retrospective chart review	10 patients (20 breasts) for gender-affirming mastectomy, 7 patients (14 breasts) for NSM with autologous reconstruction	17.5 years (range 16–19) for gender-affirming group; 49 years (range 35–62) for NSM group	Nipple neurotization in gender-affirming mastectomy and nipple-sparing mastectomy (NSM) with autologous breast reconstruction	Semmes-Weinstein monofilament testing	Significant improvement in nipple, areola, and peripheral breast skin sensation in neurotization group vs. control (*P*=0.0001); sensation loss statistically significant in control group but not in neurotization group	NAC neurotization significantly improves sensory outcomes in gender-affirming mastectomy and NSM with autologous breast reconstruction; further studies needed on patient satisfaction and long-term outcomes
Remy et al. [30]	2024	Prospective cohort study	25 patients (50 mastectomies)	23 (IQR 20–31)	TNR in gender-affirming double incision mastectomy with free nipple grafting	Monofilament testing, quantitative sensory testing, patient-reported outcomes	NAC sensation was significantly worse at 1 month (*P* < 0.01), comparable to baseline at 3 months (*P* > 0.05), and significantly better at 12 months (*P* < 0.05); 88% of patients regained some erogenous sensation	TNR allows for restoration of NAC and chest sensation within 3 months; direct coaptation of ≥2 nerves yielded best outcomes
Rochlin et. al [36]	2020	Prospective Cohort	10, (10 control patients)	17.5 (range 16-19)	TNR in gender-affirming mastectomy	Semmes-Wienstein monofilaments compared to a cohort of patients who underwent mastectomy without neurotization	Treated patients had significant improvement sensation at the nipple (*P* ≤ 0.0002), areola (*P* = 0.0001), and peripheral breast skin (*P* = 0.0001).	Immediate re-innervation of the NAC after mastectomy enhances recovery if NAC sensation in patients undergoing female to male mastectomy
Peled et. al [4]	2023	Prospective Cohort	47	46.3 (range 17-65)	TNR in nipple-sparing mastectomy and implant-based breast reconstruction	Baseline sensitivity scores using Acroval PSSD which evaluated for one-point static and one-point moving thresholds in 5 different locations of the breast	1PM pressure thresholds included the following predictors: time (*P* < 0.0001), age (*P* = 0.004), baseline moving pressure (*P* = 0.0076), and location (*P* < 0.0001), as well as the interaction of age and time (*P* < 0.0001), baseline pressure and time (*P* < 0.0001), and location and time (*P* = 0.0622); 1PS pressure thresholds were also analyzed in the same mixed effects model with time (*P* < 0.0001)	6 months post-op 80% of women had good to excellent one point 1PM and 1PS testing averaged across all areas tested, no dysesthesia or neuroma. This approach allows for preservation of high degrees of breast and NAC sensation in most patients
Zhang et al [33]	2024	Retrospective Study	58 patients but only 4 underwent neurotization	45.5, + 9.6	TNR in implant-based breast reconstruction	Semmes Wilson Monofilament testing, and the Breast Q-Sensation Module was completed	Diminished sensation across all regions 3 months post op, with significant improvement between each post op time point up to 12 months, most patients reported improve sensation over time with no dysesthesia or chronic pain	There was a significant improvement in sensory monofilament values in all 4 breast quadrants and the NAC between 3-6 months and 6-12 months post op
Tevlin et. al [28]	2021	Retrospective Chart Review	14	49 (range 32-61)	TNR in immediate autologous breast reconstruction	Sensory outcomes with the Semmes-Weinstein monofilament compared to breasts that did not undergo neurotization	No statistically significant difference in sensation between pre-op and post op nipple sensation at final follow up	Immediate re-innervation of the NAC in the setting of immediate breast reconstruction enhances recovery of the NAC sensation
Djohan et. al [3]	2020	Prospectively Mainted Cohort but data was reviewed retrospectively	8 patients with 15 breasts	38.12 + 7.5 years	TNR in implant-based breast reconstruction	Using a pressure-specific sensory device, 15 breasts underwent one post op sensation test, only 5 did 2. or sensory measurement. Measured static and dynamic pressure sensation	The nipple had a mean threshold of 67.33 ± 34.48 g/nm2. The upper inner (29 ± 26.75 g/nm2) and upper outer (46.82 ± 32.72 g/nm2) nipple-areola complex quadrants demonstrated better scores during the moving test compared with the static test.	All NAC areas had improved thresholds after the second test (10.59 + 3.75) months later
Chang et. al [37]	2024	Retrospective case control	56 underwent neurotization (positive control was 71 contralateral breasts), 16 patients with 16 breasts = negative controls	46.3+7.7	TNR in breast reconstruction	Sensory outcomes assessed every 6 months for 2 years using the Modified Research Council Sensory Scale, monofilament testing, and Tinel’s sign but monofilament testing was the primary point of analysis	Breasts that underwent neurotization had significantly better sensation after surgery, with a mean(s.d.) value of 35.61(92.63) g (*P* < 0.001). The mean(s.d.) sensory return after neurotization was gradual; 138.17(143.65) g in the first 6 months, 59.55(116.46) g at 7-12 months, 14.54(62.27) g at 13-18 months, and 0.37(0.50) g at 19-24 months after surgery.	Using the LC branch of the intercostal nerve as the innervating stump and elongating it, restores sensation after mastectomy and effectively innervates reconstructed breasts and spares the nipple/skin
Chirappapha et al [27]	2018	Prospective study	52 patients (55 NSMs)	43 years (range 31–59)	Nipple-sparing mastectomy (NSM) with implant or autologous reconstruction	Pinprick sensation test	At 6 months: 44% had partial sensation recovery, 4% had full sensation recovery; At 12 months: 60% had partial recovery, most residual sensation was in the lower areola	NSM is technically feasible with some preservation of NAC sensation, though most patients experience diminished sensitivity
Das et al. [32]	2024	Retrospective cross-sectional analysis	29 gender-affirming mastectomy cases (11 with neurotization)	Not specified	Direct neurotization of intercostal nerves to NAC during double-incision mastectomy with free nipple grafts	Operative time, work relative value units (wRVUs), and Medicare physician fee schedule values	Mean operative time: 100.3 min (without neurotization) vs. 154.2 min (with neurotization) (*P* < 0.001); Efficiency: 0.23 wRVUs/min (without neurotization) vs. 0.24 wRVUs/min (with neurotization); Difference of 0.01 wRVUs/min	Neurotization increases operative time but is appropriately valued within the Medicare reimbursement system. It is financially sustainable and could improve patient sensation and satisfaction

### TNR in Gender-Affirming Mastectomy

**Fig. 2:**
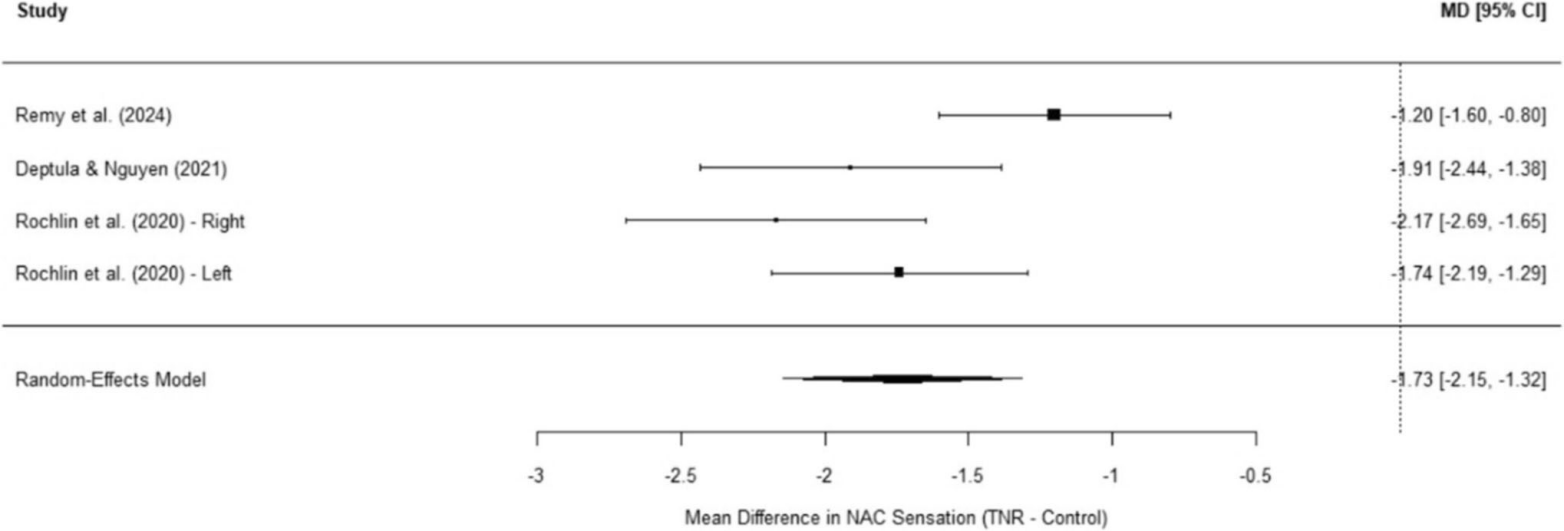
Forest plot of nipple sensation post-TNR meta-analysis

A random-effects meta-analysis was conducted on four studies evaluating monofilament thresholds for the NAC following (TNR in gender-affirming mastectomy). The pooled mean difference (MD) was  −  1.73 (SE = 0.21, 95% CI: − 2.15 to − 1.32, *p* < 0.0001), indicating a statistically significant improvement in NAC sensation among patients who underwent neurotization. The test for heterogeneity was significant (Q(3) = 9.63, *p* = 0.022), with an I^2^ of 67.78%, suggesting moderate-to-high variability across studies, likely due to differences in surgical technique, neurotization approach, and assessment tools (Figure 2).

**Fig. 3:**
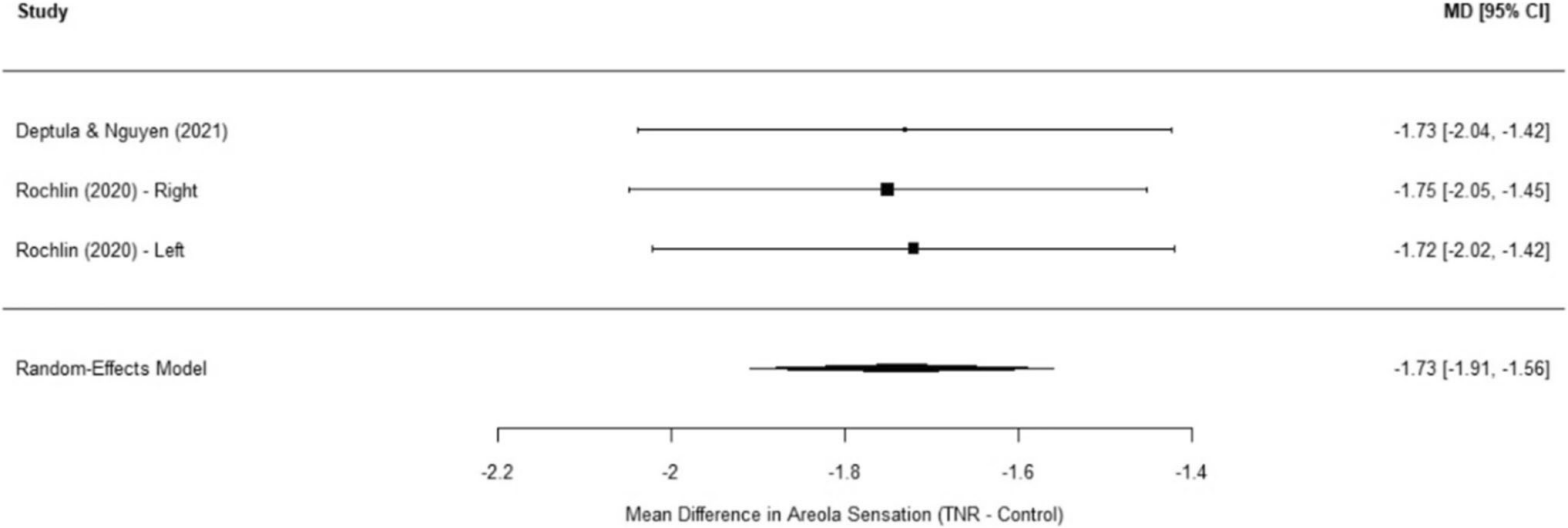
Forest plot of areola sensation post-TNR meta-analysis

**Fig. 4:**
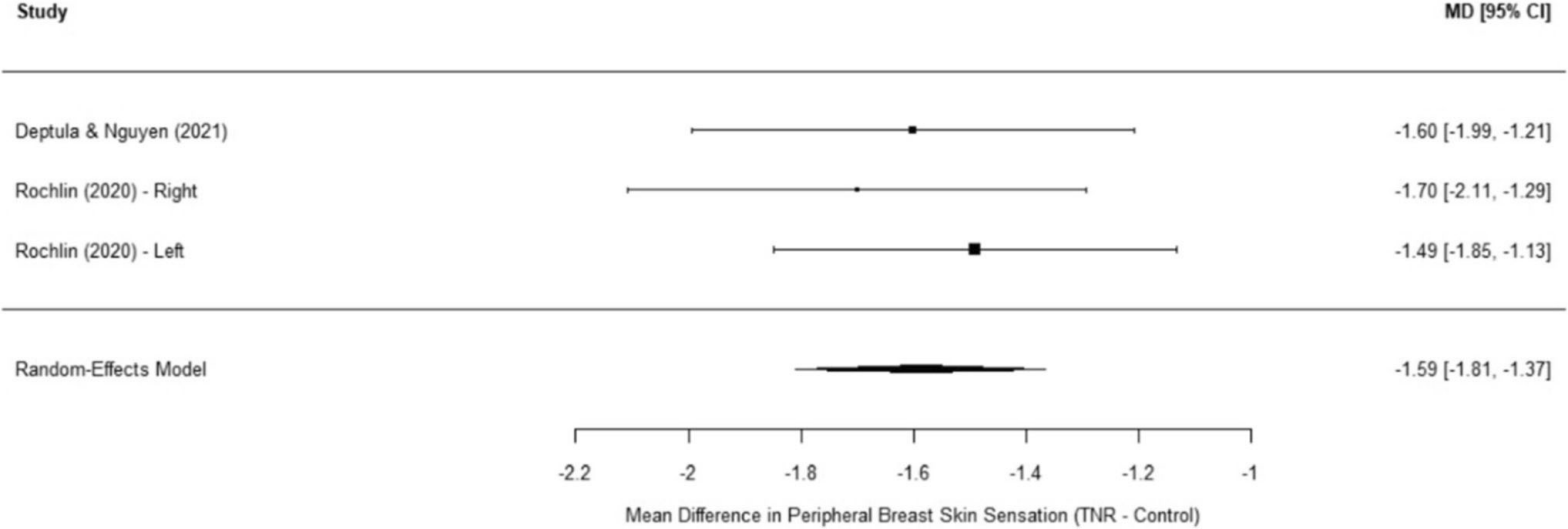
Forest plot of peripheral breast skin sensation post-TNR meta-analysis

In addition to NAC sensation, a separate meta-analysis focusing on areola sensation in gender-affirming mastectomy across three studies demonstrated a pooled mean difference of − 1.73 (SE = 0.09, 95% CI: − 1.91 to − 1.56, *p* < 0.0001) (Figure 3). Notably, no significant heterogeneity was observed (I^2^ = 0%, Q(2) = 0.02, *p* = 0.99), suggesting highly consistent findings across studies. These results further support the predictable and reproducible improvement in areola sensation following TNR. Similarly, analysis of peripheral breast skin sensation revealed a pooled mean difference of − 1.59 (SE = 0.11, 95% CI: − 1.81 to − 1.37, *p* < 0.0001), again favoring TNR. Minimal heterogeneity was noted (I^2^ = 0%, Q(2) = 0.58, *p* = 0.75), reinforcing that TNR improves sensory recovery beyond the NAC, extending to the peripheral chest region (Figure 4).

The consistency of these findings is further supported by individual studies on TNR in gender-affirming mastectomy patients. Remy et al. (2024) conducted a prospective matched cohort study that showed TNR significantly restored NAC sensation at 12 months, with patients achieving improved mechanical, temperature, and pain detection (*p* < 0.001). [1] Another study by the same group found that TNR recipients reached sensory equivalence to preoperative baselines within three months, and by 12 months, 88% had regained erogenous sensation, underscoring the potential for meaningful functional recovery with targeted neurotization techniques. [30] They also reported significantly improved patient-reported outcomes (PROs) in the TNR group, including higher satisfaction with erogenous sensation and overall chest sensibility. Loughran et al. (2024) further supported these findings, demonstrating that direct neurotization of free nipple grafts led to higher rates of normal or diminished sensation over time, increasing from 58.6% within one year to 70.8% beyond one year postoperatively. [15] Deptula & Nguyen (2021), in a retrospective chart review, compared gender-affirming mastectomy and NSM with and without TNR and found that TNR significantly improved nipple, areolar, and peripheral breast skin sensation compared to controls (*p* = 0.0001), reinforcing the value of neurotization in both gender-affirming and oncologic contexts. [31] Finally, from an economic perspective, Das et al. (2024) demonstrated that while TNR increased operative time by approximately 54 minutes, its impact on relative work value unit (wRVU) efficiency was negligible, suggesting that it is both clinically beneficial and financially sustainable within gender-affirming surgical practice. [32]

### TNR in Oncologic Breast Reconstruction

Sensory recovery following TNR in oncologic breast reconstruction was more variable. Studies assessing NAC sensation post-nipple-sparing mastectomy (NSM) with TNR reported inconsistent recovery timelines. Chirappapha et al. (2018) assessed NAC sensation recovery in nipple-sparing mastectomy (NSM) patients and found that 44% of individuals had partial sensation recovery at six months, increasing to 60% by 12 months. However, the majority of residual sensation was confined to the lower areola, suggesting limited functional restoration at the nipple itself. [27] In contrast, Peled et al. (2023) evaluated NSM patients with neurotization and found that 80% achieved good-to-excellent NAC sensation at six months, with no cases of neuromas or dysesthesia, indicating that successful TNR implementation may improve outcomes. [4]

Results in autologous breast reconstruction patients were more inconsistent. Tevlin et al. (2021) found no statistically significant difference between preoperative and postoperative NAC sensation after immediate neurotization, suggesting that spontaneous reinnervation alone may be insufficient for sensory restoration. Rochlin et al. (2020), however, demonstrated that immediate reinnervation resulted in significant sensory improvement across the NAC, areola, and peripheral breast skin (*p* ≤ 0.0002, *p* = 0.0001, and *p* = 0.0001, respectively). This supports the hypothesis that active neurotization strategies enhance sensory recovery compared to passive reinnervation. [28, 36]

In implant-based reconstruction, sensory restoration timelines were prolonged, and outcomes varied depending on neurotization technique. Djohan et al. (2020) examined sensory thresholds in NSM patients undergoing TNR with processed nerve allografts. [3] All NAC areas exhibited improved sensation after a second sensory test at a mean follow-up of 10.6 months. However, wide baseline variations in sensation indicated that nerve grafting techniques influenced recovery patterns. [33] Chang et al. (2024) studied NSM neurotization in implant-based reconstruction and found significant gradual sensory return, with the largest improvements occurring at 7–12 months (mean = 59.55 g, SD = 116.46 g) and continuing up to 24 months (mean = 0.37 g, SD = 0.50 g). [37] These results highlight the prolonged nature of sensory recovery in implant-based procedures, with some patients requiring nearly two years for optimal improvement. Additional variability in outcomes was evident in studies with heterogeneous patient populations and nerve grafting approaches. Zhang et al. (2024) examined TNR in NSM patients and found that only four of 58 patients underwent neurotization, limiting conclusions regarding efficacy. However, sensory monofilament values improved significantly across all four breast quadrants and the NAC from 3–6 months to 6–12 months postoperatively. [3] They also incorporated the BREAST-Q Sensation Module and noted that most participants reported progressive improvement in perceived sensation over 12 months, aligning with objective monofilament testing.

Overall, TNR in oncologic breast reconstruction appears to be beneficial but is less predictable compared to gender-affirming mastectomy. Factors contributing to this variability include older patient age, differences in nerve coaptation techniques, and variation in reconstructive approaches (implant vs. autologous tissue reconstruction).

## Discussion

This systematic review and meta-analysis provide robust evidence supporting the efficacy of targeted neurotization (TNR) in restoring nipple-areola complex (NAC) sensation following mastectomy.

### Sensory Recovery Following TNR in Gender-Affirming Mastectomy

The data consistently indicate that TNR in gender-affirming mastectomy leads to early and substantial sensory restoration. [2, 12–15, 30, 34] Meta-analyses showed that NAC, areola, and peripheral breast skin sensation significantly improved following TNR (*p* < 0.0001 for all measures). Notably, some studies reported that patients regained preoperative sensory baselines within three months, with 88% experiencing restored erogenous sensation by 12 months. [1] This rapid recovery is likely attributable to the surgical prioritization of nerve preservation and coaptation, ensuring direct neurotization at the time of mastectomy. Additionally, gender-affirming patients are typically younger (mean age: 20s to 30s), which may contribute to enhanced nerve regeneration and plasticity. These patients also tend to have different expectations and motivations, with sensory function often regarded as an essential postoperative outcome alongside aesthetic considerations. The absence of neuroma formation or dysesthesia in these studies further supports TNR as a safe and effective intervention in gender-affirming procedures. Additionally, economic modeling by Das et al. (2024) suggests that while TNR increases operative time, it has minimal impact on wRVU efficiency, making it a financially sustainable intervention. [32]

### Sensory Recovery Following TNR in Oncologic Mastectomy

While TNR also improved sensory outcomes in oncologic mastectomy, recovery was often delayed and less predictable compared to gender-affirming cohorts. Studies on NSM reported only partial sensory restoration at 6–12 months, with residual sensation often limited to the lower areola rather than the nipple itself. [27] Although Peled et al. (2023) found that 80% of patients achieved good-to-excellent sensation by six months postoperatively, other studies, such as Tevlin et al. (2021), reported no significant difference between preoperative and postoperative NAC sensation, highlighting inconsistencies in outcomes. [4, 28] The primary reason for this variability lies in the surgical priorities of oncologic mastectomy. Unlike gender-affirming mastectomy, which emphasizes functional and sensory preservation, oncologic mastectomy prioritizes complete oncologic clearance. This often results in greater nerve disruption and unpredictability in spontaneous reinnervation. Furthermore, oncologic patients tend to be older (mean age: 40s to 50s), which may limit nerve regeneration potential. Standardization of neurotization techniques, particularly in NSM and immediate breast reconstruction, is necessary to improve outcomes in this population.

### Comparison of Gender-Affirming vs. Oncologic Mastectomy Outcomes

A direct comparison between gender-affirming and oncologic mastectomy highlights key distinctions in sensory recovery following TNR. Gender-affirming patients consistently exhibited faster and more uniform improvements in NAC sensation, whereas oncologic patients demonstrated more heterogeneity and prolonged recovery timelines. These disparities likely stem from differences in nerve preservation strategies, patient age, and the role of oncologic clearance in surgical planning. Despite these differences, the effectiveness of TNR across both groups supports its integration into reconstructive protocols. Given its potential to enhance patient satisfaction and functional outcomes, future research should focus on optimizing neurotization techniques, standardizing outcome assessment measures, and exploring long-term sensory restoration beyond 12 months. In addition, PROs also demonstrated meaningful gains in both groups. Remy et al. highlighted enhanced patient satisfaction and erogenous sensibility following TNR in gender-affirming mastectomy, while Zhang et al. corroborated these findings in oncologic reconstruction using the BREAST-Q Sensation Module. These subjective metrics affirm the functional and psychosocial relevance of sensory restoration, reinforcing the value of TNR beyond anatomical outcomes.

### Expanded Consideration: Thermal vs. Tactile Sensory Recovery

In interpreting these sensory outcomes, it is important to distinguish between different sensory modalities. Most studies in this review emphasized tactile sensation, assessed using tools such as monofilament testing. However, thermosensation—the ability to perceive heat and cold—plays a crucial protective role, particularly in reconstructed breasts that lack native innervation. This distinction has critical clinical implications in oncologic populations, where diminished thermal perception can increase the risk of unrecognized burns or thermal injury. The integration of thermal testing alongside mechanosensory evaluations may better capture the functional relevance of sensory recovery and guide patient counseling and postoperative safety precautions.

### Future Directions and Limitations

This study highlights several areas for further investigation. First, there is substantial heterogeneity in neurotization techniques and outcome assessment tools across studies, making direct comparisons challenging. While most studies utilized monofilament testing, variations in donor nerve selection, coaptation strategies, and follow-up duration necessitate the development of standardized protocols. The inclusion of validated patient-reported outcome measures (PROMs), such as the BREAST-Q Sensation Module, would enhance the evaluation of sensory recovery and patient satisfaction. [35] Zhang et al. employed this module in oncologic patients and reported meaningful improvements in self-reported sensation and satisfaction, highlighting the psychosocial importance of TNR beyond objective thresholds. Additionally, the long-term durability of TNR remains uncertain, as most studies report follow-up limited to 12 months. Further research is needed to assess whether sensory improvements persist beyond the first postoperative year and whether additional interventions, such as secondary neurotization procedures, could further enhance outcomes. [13] Lastly, while gender-affirming mastectomy patients demonstrate superior early sensory recovery, the broader psychosocial impact of NAC sensation restoration remains underexplored. For gender-affirming patients, in particular, the return of protective and pleasurable sensation plays a critical role in fostering embodiment, identity affirmation, and intimate relationships. Future studies should incorporate both quantitative sensory testing and longitudinal PROs to evaluate how TNR contributes to the lived experience of postoperative recovery.

### Clinical Integration and Applicability

The findings of this review underscore the feasibility of integrating TNR into routine surgical practice, particularly during gender-affirming and select oncologic mastectomies. Intraoperative identification and preservation of the lateral intercostal nerves can be achieved with standard techniques, especially in double-incision or nipple-sparing approaches. Notably, studies such as Rochlin et al. and Loughran et al. show that TNR can be implemented outside academic centers with minimal additional resources. Surgeon training in nerve coaptation—via cadaveric labs or intraoperative mentorship—and institutional support are essential for broader adoption. Das et al. demonstrated that TNR adds ~54 minutes of operative time but has minimal impact on wRVU efficiency, supporting its economic viability. Moving forward, implementation should focus on standardizing coaptation techniques and outcome measures, incorporating TNR training into surgical education, and fostering multidisciplinary collaboration in oncologic settings where oncologic safety must be balanced with sensory restoration. As evidence and demand grow, structured protocols and equitable access will be key to making TNR a routine component of breast surgery

## Conclusion

TNR significantly enhances NAC sensation in both gender-affirming and oncologic mastectomy patients, though gender-affirming cohorts demonstrate more rapid and consistent recovery. Oncologic mastectomy outcomes remain variable, likely due to differences in surgical priorities and patient demographics. Despite these discrepancies, the clinical and economic feasibility of TNR supports its integration into both gender-affirming and oncologic reconstructive protocols. Future research should focus on refining neurotization techniques, standardizing outcome measures and PROs , and evaluating long-term sensory restoration to optimize patient outcomes across all mastectomy populations.
